# Reported Effectiveness of a Text‐Based Post‐Operative Care Intervention After Voluntary Medical Male Circumcision (VMMC) to Improve Quality of Care and Adverse Events Identification in Sub‐Saharan Africa: A Scoping Review

**DOI:** 10.1002/hsr2.72752

**Published:** 2026-07-04

**Authors:** Calsile Makhele, Evans Duah, Smangaliso Morerwa, Kuhlula Maluleke

**Affiliations:** ^1^ School of Health Systems and Public Health, Faculty of Health Sciences University of Pretoria Pretoria South Africa; ^2^ The Aurum Institute Johannesburg South Africa; ^3^ Department of Health Sciences, Centre for Development and Implementation of Point‐of‐care Diagnostics University of Pretoria Pretoria South Africa; ^4^ Library Services University of Pretoria Pretoria South Africa

**Keywords:** adverse events, mHealth, post‐operative care, text‐based follow‐up, two‐way‐texting, voluntary medical male circumcision

## Abstract

**Background and Aim:**

Although the two‐way texting (2wT) intervention is a promising mobile‐health (mHealth) approach, limited research exists on its implementation, acceptability and potential benefits within Voluntary Medical Male Circumcision (VMMC) context. This scoping review mapped the available literature on 2wT's application in VMMC post‐operative care, focusing on outcomes related to quality of care and adverse event identification in Sub‐Saharan Africa. These findings provide a foundation for healthcare providers and policymakers to consider integrating digital health platforms into VMMC programs and highlight areas for further research to inform evidence‐based decisions and optimize service delivery.

**Methods:**

To consolidate the evidence, we reviewed literature published between 2010 and 2025 following the advanced methodological framework of Arksey and O'Malley, Levac et al., and PRISMA‐ScR guidelines. Comprehensive searches were conducted in Scopus, Web of Science, ProQuest, EBSCOhost (MEDLINE, CINAHL) databases, as well as relevant grey literature sources. Two independent reviewers screened the data using Covidence, and methodological quality was appraised using the Mixed Methods Appraisal Tool (MMAT) version 2018.

**Results:**

From the 14 studies included out of 2869 studies screened, findings were synthesized and presented under formulated themes. All articles had MMAT scores between 70% and 100% which proves average to high methodological quality.

**Conclusion:**

Our review indicates that the 2wT approach is associated with improved patient follow‐up adherence, adverse event detection, and potential enhancements in health system efficiency in VMMC post‐operative care.

## 1 Introduction

Voluntary Medical Male Circumcision (VMMC) upscaling faces a number of challenges in sub‐Saharan Africa (SSA) countries, however, it is still recognised as a key strategy in HIV prevention and in reducing female‐to‐male HIV transmission by approximately 60% [1, 2, 3, 4]. Since its large‐scale implementation in 2007, nearly 29.6 million procedures have been performed, preventing an estimated 615,000 new HIV infections by 2020 [4, 5]. Countries in SSA with high HIV prevalence, including South Africa and Zimbabwe, have adopted VMMC widely [1]. Over 35 million voluntary medical male circumcisions (VMMCs) have been performed in the Joint World Health Organization (WHO) and United Nations Program on HIV/AIDS (UNAIDS) priority countries, with significant progress reported across the 15 priority countries in Eastern and Southern Africa [6, 7]. However, COVID‐19 significantly impacted service delivery [8, 9], which resulted in decreased VMMC uptake globally between 2020 and 2021 [10, 11]. WHO and partner organizations responded by advocating for resilient service delivery approaches, which include integrating VMMC intervention into standard health services and using digital tools for follow‐up [12].

WHO outlined the VMMC Quality Assurance (QA) [13] scale‐up guide which emphasizes the quality of services encompassing HIV and VMMC education, HIV testing and counselling, safe surgical procedures, post‐operative instructions and recovery, and at least two post‐operation follow‐ups [14, 15, 16]. Through the identification, management, and treatment of surgical post‐operation complications, regular routine follow‐up visits contribute to patient safety [17]. These guidelines are intended to streamline the VMMC procedure and maximize its efficiency [13]. However, because of its many components, VMMC in low‐resourced countries faces challenges such as staff shortages [18], low adherence to follow‐up visits due to transportation barriers [19], concerns about privacy during clinic‐based post‐operative care, and limited access to healthcare [20, 21, 22].

Post‐operative care is essential for patient recovery and identification of adverse events (AEs) [17]. Poor adherence to post‐operative care increases the risk of avoidable problems, including delays in detecting adverse events that could otherwise be controlled with prompt management [23, 24]. In order to prevent provider attrition and maximize performance, it is also crucial that efficiency considerations be contextualized appropriately, particularly in settings with limited resources [25]. This is because increasing VMMC upscale puts additional strain on staffing, causing work fatigue and burnout [18, 26, 27]. However, mobile health interventions, specifically 2wT, have demonstrated potential to facilitate patient‐provider communication, improving follow‐up adherence and AE detection [15, 21, 28].

The rapid expansion of digital health technologies presents new opportunities for overcoming these challenges [9]. In African countries, digital innovation is playing a transformative role in HIV prevention by providing novel solutions that enhance service delivery and patient engagement [29]. SSA's increasing technology and mobile phone ownership rate of about 67% presents a chance to use technology to close these gaps and advance the UNAIDS 95‐95‐95 targets [30].

mHealth interventions, particularly text‐based communication tools, have demonstrated potential in facilitating patient‐provider interactions [29, 31]. 2wT provides real‐time communication between patients and healthcare providers which enables correctly informed medical guidance, that may help bridge the gaps in post‐operative VMMC care [21, 32, 33]. Digital health emerged as a critical strategy for improving health system performance, particularly in low‐ and middle‐income countries (LMICs) [34]. Within this domain, mobile health offers promising avenues to overcome infrastructural and human resource limitations [34]. In recognizing the potential of digital innovation, the WHO introduced the Global Strategy on Digital Health 2020–2025 [35], which advocates for scalable, evidence‐based digital solutions integrated into national health strategies [36].

Despite growing evidence supporting mHealth interventions [37, 38], empirical research on the adoption, feasibility and reported effectiveness of 2wT in VMMC programs remains limited and heterogeneous, particularly in sub‐Saharan Africa. Existing studies vary in study design, setting and geographic focus, making it difficult to draw consistent inferences. A scoping review is therefore warranted to map the existing literature on 2wT intervention, including its reported effectiveness and feasibility in VMMC programs. This review also seeks to identify gaps and challenges in 2wT adoption and to provide evidence‐informed recommendations for its integration and potential scale‐up within routine VMMC care in sub‐Saharan Africa.

## 2 Methods

### 2.1 Study Design

This scoping review followed the Arksey & O'Malley framework [39], refined by Levac et al. [40], and reported according to Preferred Reporting Items for Systematic Reviews and Meta‐Analyses extension for Scoping Reviews PRISMA‐ScR guidelines [41]. This study followed 5 key stages: i) defining the research topic; ii) finding relevant articles; iii) choosing eligible articles; iv) charting the data; v) collating, summarizing, and reporting the results. This scoping review is registered on the Open Science Framework and is accessible via: https://osf.io/mfk2z.

### 2.2 Defining the Research Topic

The research question was: *What is the reported effectiveness of 2wT mHealth interventions in improving post‐operative care quality and early AE identification in VMMC?*

This review focused on sub‐Saharan African countries where text‐based follow‐up systems for VMMC clientele have been implemented to some degree, and used the population, concept, and context (PCC) framework as shown in Table 1 for determining the eligibility of the research question.

**Table 1:** Population, concept, context.

Population	Patients undergoing Voluntary Medical Male Circumcision
Concept	2wT/Mobile health interventions for post‐operative care
Context	Healthcare facilities in sub‐Saharan Africa

### 2.3 Finding Relevant Articles

The databases searched included: EBSCOhost: (MEDLINE, CINAHL, Health Source: Nursing/Academic Edition, Health Source ‐ Consumer Edition, Academic Complete, Africa‐Wide Information), Scopus, PubMed, Web of Science and some of the searching key terms used were:

(“Voluntary Medical Male Circumcision” OR VMMC OR “male circumcision” OR “medical circumcision” OR “circumcised men” OR “HIV prevention” OR “Circumcised males”)

AND (“2‐way texting” OR “two‐way texting” OR 2wT OR “interactive SMS” OR “mobile texting” OR “text messaging” OR mHealth OR “mobile health” OR “SMS follow‐up” OR “text‐based follow‐up” OR “digital health” OR “Interactive messaging” OR “Patient engagement” OR “Follow‐up system” OR “Text‐based telehealth”) and were combined with Boolean operators. Grey literature included WHO, UNAIDS, and 2wT project reports. Individual search strings and outputs are presented as supporting material 1.

### 2.4 Choosing Eligible Articles

#### 2.4.1 Inclusion Criteria

Records were included if they:

- Provide evidence on the use of 2wT or mHealth interventions in healthcare.
- Specifically focus on VMMC programs or include VMMC post‐operative care.
- Assess effectiveness, feasibility, or implementation of 2wT/mHealth for post‐operative follow‐up or early detection of adverse events.
- Provide outcomes of interest including patient follow‐up adherence, adverse events identification and management, health resources optimization and outcomes, acceptability and usability of the intervention, and cost‐related metrics.
- Are conducted in sub‐Saharan Africa.
- Include empirical data from quantitative, qualitative, or mixed‐methods studies.
- Are published in peer‐reviewed journals or as relevant grey literature (e.g., government reports, WHO/NGO publications).
- Are published between 2010 and 2025 to reflect the period of increasing mHealth adoption.
- Are available in English or can be translated into English.

#### 2.4.2 Exclusion Criteria

Records were excluded if they:

- Do not include 2wT or fail to distinguish 2wT from other forms of mHealth.
- Focus only on one‐way messaging or non‐interactive communication methods.
- Do not involve post‐operative follow‐up care or adverse event detection in VMMC.
- Are conducted exclusively in high‐income countries and outside sub‐Saharan Africa.
- Are editorials, opinion pieces, commentaries, or conference abstracts.
- Address digital health interventions unrelated to VMMC or unrelated to patient follow‐up.
- Lack a clear study design or documented outcomes related to quality of care or AE identification.

**Figure 1:**
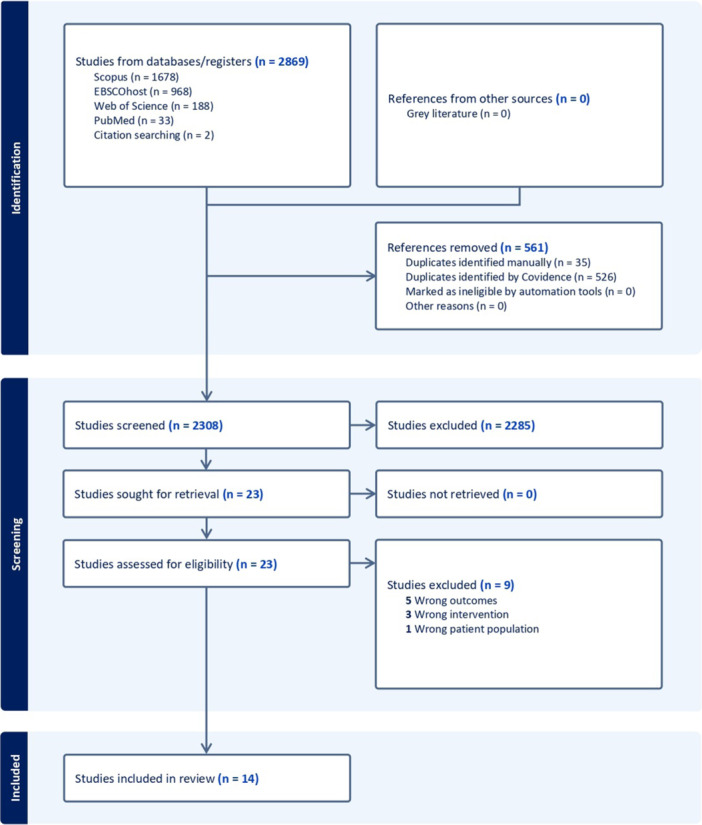
PRISMA‐ScR flowchart showing screening process and eligible literature.

Two reviewers independently screened the literatures first by titles and abstracts, then by reviewing full texts of each article. Inter‐rater agreement was assessed using Cohen's kappa statistic: 0–0.1 = no agreement, 0.11–0.20 = slight, 0.21–0.40 = fair, 0.41–0.60 = moderate, 0.61–0.80 = substantial, 0.81–1.00 = almost perfect [42]. The study selection process is illustrated in the PRISMA‐ScR flow diagram (Figure 1). All discrepancies at the title and abstract screening were resolved unanimously through discussion. However, a third screener resolved all conflicts arising from the full text screening.

### 2.5 Charting the Data

A standardized charting table was employed to capture key study characteristics, including author and year of publication, journal, study aim, study population, study setting (rural or urban), study location, study design, and the type of text‐based post‐operative intervention, the main findings and other significant findings from each study.

### 2.6 Collating, Summarizing, and Reporting the Results

The Mixed Methods Appraisal Tool (MMAT) version 2018 was used to assess study quality [43]. Scores were graded as: ≤ 50% = low, 51–75% = average, 76–100% = good quality. Two reviewers independently appraised studies; discrepancies were resolved by a third reviewer. Given the heterogeneity of study designs and outcomes, no pooled quantitative synthesis was performed, and no causal inferences were drawn, as scoping reviews are not intended to determine causality. Instead, extracted data were narratively summarized and thematically analysed, generating the following themes presented in the results: reported effectiveness of 2wT in post‐operative care and adverse event management; acceptability and usability for clients and healthcare workers; reported cost‐effectiveness and efficiency of 2wT; and implementation and scalability challenges. This approach ensured that all relevant evidence was systematically captured while accommodating the diversity of outcomes and study designs included in this scoping review.

### 2.7 Ethical Consideration

No primary data collection was performed; however, ethical clearance was obtained from the Faculty of Health Sciences Research Ethics Committee, University of Pretoria: 493/2025.

## 3 Results

### 3.1 Screening

The database searches identified 2869 articles for screening, 561 duplicates were removed, 2285 were excluded through title and abstract screening and 23 met the inclusion criteria for full‐article screening (Figure 1). During full‐text screening, nine articles were excluded for various reasons. Five studies were excluded due to wrong study outcomes; these were studies that assessed outcomes unrelated to text‐based or digital communication interventions (e.g., surgical techniques or clinical efficacy of circumcision procedures) or reported general health outcomes without linking them to a text‐based post‐operative care strategy. Three studies were excluded due to wrong interventions, as they evaluated interventions other than 2wT. One study was excluded due to the wrong patient population, as it involved participants who did not meet the defined eligibility criteria for this review. Fourteen articles met the inclusion criteria and were included in the review. The independent reviewers had a moderate agreement (Cohen's Kappa statistic = 0.55).

### 3.2 Characteristics of Included Studies

**Figure 2:**
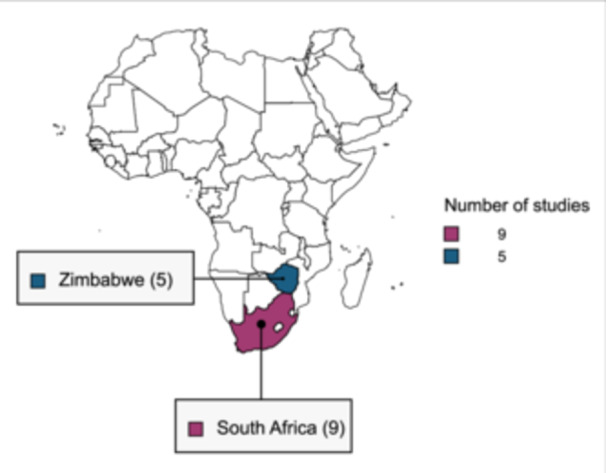
A map showing a geographic distribution of published evidence.

All included studies were characterised and summarized in Supporting Material 2. These studies included articles published between 2010 and 2025. Studies were geographically mapped to show which countries implemented the intervention and have most of the evidence on effectiveness of a Text‐Based Post‐Operative Care Intervention after Voluntary Medical Male Circumcision (VMMC) to Improve Quality of Care and Adverse Events Identification in Sub‐Saharan Africa. All the included studies were conducted in South Africa and Zimbabwe (Figure 2).

### 3.3 Quality of Evidence

The 14 eligible studies were appraised using the MMAT checklist (supporting material 3), for methodological quality scored as follows: high‐quality studies (76%–100%), average‐quality (51%–75%) and low‐quality (≤ 50%) [43]. Only peer‐reviewed studies met the eligibility criteria. Five studies scored 70%–75% indicating average methodological quality [33, 44, 45, 46, 47] whereas nine scored 80%–100% indicating high quality [15, 21, 48, 49, 50, 51, 52, 53, 54]. This distribution indicates that findings from the high‐quality studies, particularly objective outcomes such as reductions in clinic visits, adverse event detection, and follow‐up adherence, can be interpreted with relatively high confidence. In contrast, outcomes reported primarily in average‐quality studies, such as user acceptability, usability, and perceptions of the 2wT system, should be interpreted with caution.

### 3.4 Summary of Findings

The following themes emerged from the review: reported 2wT effectiveness in post‐operative care and AE management, acceptability and usability for clients and healthcare workers, reported cost effectiveness and efficiency of 2wT, and implementation and scalability challenges and enablers.

### 3.5 Reported 2wT Effectiveness in Post‐Operative Care and AE Management

Ten of the 14 studies that implemented 2wT in Zimbabwe and South Africa reported its effectiveness by reducing post‐circumcision physical visits, with some studies reporting decreases of approximately 60%–80% compared with routine clinic‐based follow‐up schedules [15, 21, 44, 46, 47, 48, 49, 51, 52, 53]. Feldacker et al. reported that 2wT follow‐up produced AE identification rates comparable to standard in‐person care, with 2.3% of 2wT clients and 1% of routine care clients experiencing AEs [21]. Babigumira et al. also reported that 2wT improved AE ascertainment by early identification of 92% of AEs compared with 42% under routine care [48]. The 2wT approach substantially reduced the burden of in‐person follow‐up, with the average number of visits decreasing from 1.34 in routine care to 0.22 in the 2wT arm [21]. Overall, 84.8% of 2wT participants did not attend any in‐person postoperative visits, compared with 19.6% in the routine care group [21], demonstrating that the text‐based system effectively maintained patient monitoring while minimizing clinic visits.

These findings support the effectiveness of 2wT as a high‐volume, text‐based post‐VMMC follow‐up method while maintaining quality of care. It should be noted, however, that safety in terms of serious or delayed AE presentations was not explicitly reported, indicating a limitation in the current evidence and highlighting the need for ongoing monitoring in large‐scale implementation.

### 3.6 Acceptability and Usability for Clients and Healthcare Workers

The text‐based system users shared their experiences through few quantitative and qualitative studies and reported their perceived acceptability and usability of the 2wT intervention [54]. In qualitative studies, both clients and healthcare workers reported that the 2wT approach was easy to use and allowed patients to maintain privacy, particularly for those who did not want to be seen repeatedly visiting the clinics [15, 45, 49, 50]. Although a few studies noted that the texting system was available in only a limited number of languages, restricting access for some clients, it did allow clients to send messages even outside of working hours [49, 53, 55]. The 2wT texting platform, operated through the open‐source Community Health Toolkit (CHT) and linked to a dashboard [49, 51, 54], recorded trends such as limited language options, poor client attendance, after‐hours workloads for HCWs, and poor follow up rates [52]. High acceptability among the healthcare workers and clients was consistently reported, largely due to the convenience, privacy, and health education empowerment provided by text instructions [15, 45, 49, 50, 54]. User‐centered design and language adaptation were additional themes that enhanced engagement between nurses and clients post‐VMMC procedures [15, 45].

Overall, these findings suggest that while 2wT is highly acceptable and supports patient engagement and wound care education, successful implementation requires addressing language accessibility, staff workload, and follow‐up challenges to maximize its impact.

### 3.7 Reported Cost‐Effectiveness and Efficiency of 2wT

Depending on the scale‐up and coverage of the implementation and in rural‐urban context, three studies evaluating the cost‐effectiveness of 2wT reported savings ranging from US$0.29 and US$3.56 per client, reflecting improvements compared to routine follow‐up costs [46, 47, 48]. Post VMMC clients reported that 2wT reduces both time and expense, as clients spend less on travel and healthcare workers have fewer follow‐up visits, allowing them to allocate more time to other procedures. Across the three studies, 2wT was compared with routine post‐VMMC follow‐up, which involved scheduled in‐person visits and active tracing for missed appointments.

In all studies, 2wT replaced most clinic visits with SMS‐based follow‐up, enhanced post‐operative counselling, and nurse‐led triaging of potential adverse events. Costing approaches differed across studies: Su et al. and Babigumira et al. primarily captured operational and marginal costs, such as nurse time, messaging, and follow‐up activities, but largely excluded fixed costs, system setup, training, and long‐term maintenance [46, 48]. Unsworth et al. incorporated personnel, SMS platform maintenance, capital equipment (annualized), and additional staff time during high‐volume periods, providing a more comprehensive estimate of routine implementation costs [48]. Across settings, 2wT consistently reduced costs by limiting unnecessary clinic visits and tracing, with greater savings observed in rural areas where programs covered transportation costs.

However, caution is warranted in interpreting these findings. Differences in costing methods, assumptions about tracing activities, and partial inclusion of system‐level costs mean that the magnitude of savings may vary across contexts. Findings from Unsworth et al. are likely the most generalizable to routine, scaled‐up programs, as they reflect a full accounting of both recurrent and capital costs, whereas savings reported in trial‐based studies may be inflated by trial‐specific conditions. Overall, the findings suggest that 2wT is a cost‐saving and efficient alternative to routine follow‐up, particularly in rural or resource‐limited settings, but careful consideration of local infrastructure and scale‐up requirements is necessary when extrapolating results.

### 3.8 Implementation and Scalability Challenges and Enablers

Implementation and scalability challenges and enablers associated with 2wT were reported by six of the included studies [21, 28, 33, 50, 51, 54]. The results highlighted common challenges including understaffing, inadequate policy frameworks, limited digital infrastructure, low client digital literacy, especially in remote areas; employee's negative attitudes towards technology‐based systems, and the absence of clear national standards for adopting digital health [21, 28, 33, 50, 51, 54]. Despite these barriers, successful implementation was supported by collaboration among 2wT technology developers (Medic mobile), non‐governmental implementing partners, and Ministries of Health. For example, the Zimbabwe's AIDS Intervention Consortium (ZAZIC) program used a structured three‐tier scale‐up approach with feedback loops and monitoring dashboards to transition 2wT from a pilot intervention to national programmatic implementation [20, 27]. This suggests that a coordinated implementation, government engagement, and attention to digital platform interoperability may support successful scale‐up [44, 49]. Finally, to ensure a long‐term integration of 2wT into VMMC programs, Setswe et al. emphasized the significance of striking a balance between research rigor and practical flexibility [33]. This balance is essential to support real‐world implementation, where rigid study conditions may limit adaptability, while excessive flexibility may compromise intervention fidelity. Maintaining this equilibrium can enhance sustainability, improve scalability, and facilitate integration of 2wT within routine service delivery in diverse implementation settings. Although this theme was not a predefined primary outcome of the review, it emerged consistently during data extraction and was therefore included as an inductive theme to inform future scale‐up of 2wT programs.

## 4 Discussion

Across Zimbabwe and South Africa, the two main 2wT implementation settings in SSA, evidence suggests that 2wT is an effective, acceptable, cost‐saving, and potentially scalable approach for post‐VMMC follow‐up, as it reduces clinic visits while maintaining reported quality of care; however, implementation success appears to depend on addressing key contextual and system‐level barriers. Overall, findings from studies in both countries suggest that 2wT is a user‐friendly approach that supports post‐circumcision wound care. This intervention complements routine guidelines by enabling triage of potential adverse events without overburdening healthcare workers [21, 28, 44, 45, 46, 49, 50, 53]. The reviewed data also suggest that the text‐based intervention may be feasible and beneficial in both rural and urban settings, particularly in low resources areas [21, 44, 46, 52, 53].

This text‐based system facilitated post‐operative care by enabling communication between clients and healthcare workers, allowing educational support and follow‐up to be provided remotely while reducing the need for routine in‐person clinic visits [21, 44, 46, 53]. Direct access to nurses via mobile phones was reported to improve efficiency of follow‐up services and support the early identification of AEs, as well as post‐operative wound management. However, the available evidence primarily reflects the system's ability to facilitate monitoring and AE detection rather than providing definitive evidence of improved patient safety or clinical outcomes. In rural and low‐resource communities, where transportation costs and long travel distances can limit access to care, 2wT may help support post‐operative follow‐up and strengthen communication between clients and healthcare providers [14, 23]. Additionally, other mHealth studies have reported that text‐based interventions may enhance patient engagement in HIV prevention and treatment programs across SSA and contribute to continuity of care [30, 32].

Patients and healthcare workers using 2wT generally reported high acceptance and satisfaction, highlighting the system's ease of use, privacy, and perceived benefits for communication and wound care management [15, 45, 54]. Users appreciated the ability to seek guidance, and report concerns without attending the clinic in person, which helped maintain confidentiality and reduce discomfort associated with frequent visits [15, 21, 44, 54]. The platform also supported health education by providing structured, clear instructions and real‐time feedback, empowering patients to take ownership of their post‐operative care. Despite strong evidence of usability and positive user experiences with 2wT, most African health systems continue to face policy, governance, and infrastructure challenges that hinder the formal integration of digital platforms into routine care [56, 57], and text‐based follow‐up has not yet been incorporated into VMMC guidelines in most countries, which may limit its adoption, scalability, and long‐term sustainability. Moreover, healthcare workers noted concerns about potential intrusions outside working hours, as clients could interact with them beyond clinic times, and about the limited language options on the platform, particularly in linguistically diverse settings such as South Africa [21, 50, 54]. Additionally, studies reported cost savings associated with 2wT, ranging from US$0.29 to US$3.56 per client, primarily due to reduced patient transport visits and lower operational staffing costs [46, 47].

Despite the reported effectiveness of 2wT, several contextual and system‐level barriers constraints its implementation and scale‐up in SSA. Key challenges include limited mobile network coverage in rural areas, inconsistent electricity supply, low digital literacy among both patients and health workers, and concerns around data privacy and security [18, 58]. In addition, weak policy and regulatory frameworks, inadequate digital health infrastructure, and negative attitudes toward technology‐based systems among some health workers further hinder implementation efforts [44, 45, 49, 50, 54]. In contrast, several enabling factors were identified. These include strong government buy‐in, integration of digital tools into existing health systems, effective public‐private partnerships, and donor and development partner support [23, 59]. Human‐centered design approaches and community engagement have also shown to improve acceptability, usability and uptake of digital health interventions [60]. Future research should explore the scalability of 2wT within national eHealth frameworks, examine long‐term cost implications, and investigate patient and provider experiences beyond research settings to inform potential large‐scale implementation [21, 46, 48].

## 5 Strengths and Limitations

A key strength of this scoping review is the comprehensive synthesis of the available evidence, conducted in accordance with the PRISMA‐ScR and Arksey and O'Malley methodological frameworks. By incorporating randomized controlled trials, qualitative studies, implementation studies, and cost‐effectiveness analyses, the review provides a broad understanding of the operational, economic, and acceptability dimensions of the 2wT intervention. The inclusion of diverse study designs, facilitated by the scoping review methodology, enabled a more comprehensive exploration of the evidence base. Furthermore, nine of the 14 included studies were rated as high methodological quality, providing reasonable confidence in findings related to objective outcomes such as follow‐up adherence, adverse event detection, and reductions in clinic visits.

Nevertheless, several limitations should be considered. Findings related to user acceptability, usability, and perceptions of the 2wT system were reported primarily in studies of moderate methodological quality, with five studies falling into this category; therefore, these findings should be interpreted with some caution. In addition, most included studies were conducted in pilot or controlled implementation settings, which may limit the applicability of the findings to routine national programmes. Another important limitation of the available evidence concerns the assessment of safety outcomes. Most studies relied on adverse event detection and ascertainment measures, which are indirect proxies and may not accurately reflect true patient safety. None of the included studies employed comprehensive safety assessment methods capable of definitively evaluating serious, delayed, or disfiguring adverse events. Consequently, conclusions regarding the safety of 2wT should be interpreted cautiously, and future studies should incorporate more robust safety outcome measures. The review also identified limited evidence on cross‐platform interoperability, long‐term sustainability, and health‐system integration. In addition, reporting gaps, including inconsistent outcome definitions, limited data on late adverse events, and insufficient representation of rural and linguistically diverse populations, may have constrained the comprehensiveness of the synthesis. Publication bias may also have influenced the findings, as successful implementation experiences are more likely to be reported in peer‐reviewed literature than grey. Furthermore, the exclusion of conference abstracts may have contributed to this bias. Although a formal risk‐of‐bias assessment was not undertaken, as this is not typically required for scoping reviews, methodological quality was assessed using the MMAT, providing a partial appraisal of potential bias across qualitative, quantitative, and mixed‐methods studies. Finally, a large proportion of the included studies were conducted by the same research group in Zimbabwe and South Africa, led by Feldacker and colleagues, which may limit the generalizability of the findings. The concentration of evidence within a small number of countries, implementation settings, and research teams means that the findings may not fully reflect the challenges, outcomes, and implementation experiences that could be observed in other SSA countries with different healthcare infrastructures, digital health capacities, policy environments, and population characteristics. Therefore, the findings should be interpreted with caution, and additional independent evaluations in diverse geographical and healthcare settings are needed to strengthen the evidence base and assess the broader applicability of 2wT in VMMC follow‐up across the region.

### 5.1 Implications for Practice

This review suggests that 2wT may serve as a useful addition to standard VMMC follow‐up care. Evidence from Zimbabwe and South Africa indicates that 2wT is associated with reductions in unnecessary clinic visits, supports identification of adverse events, and may contribute to cost savings for both implementers and patients. By potentially reducing the workload for healthcare professionals, 2wT could facilitate triaging and scheduling in‐person evaluations only, when necessary, which may be particularly relevant in health systems with limited human resources. The intervention was generally reported to have high acceptability and usability among clients and providers, especially in settings with widespread mobile phone access. Integration of 2wT into post‐operative care pathways may also support patient‐centered service delivery, enhance patient‐provider communication, and promote continuity of care in line with WHO digital health guidelines. With appropriate digital governance frameworks, staff training, and standardized reporting procedures, Ministries of Health may consider further exploring the incorporation of 2wT into national VMMC follow‐up strategies.

### 5.2 Implications for Future Research

Future studies should focus on assessing 2wT's scalability and long‐term sustainability when integrated into national digital health ecosystems. Interoperability between 2wT platforms and current electronic health information systems, long‐term cost implications beyond the immediate post‐operative period, and the efficacy of 2wT across different linguistic, socioeconomic, and rural populations are important areas that need more research. An addition of operational viability and up‐scale resource requirements study would be aided by more thorough implementation research, especially studies evaluating regular, non‐pilot deployments. Also, research focusing on implementation outcomes, such as patient and provider experiences, system use, and workload over time, may help inform the sustainability of 2wT interventions, particularly beyond initial deployment phases. In addition, further research is needed to identify strategies to optimize workload distribution and reduce after‐hours demands on healthcare workers. Lastly, in order to improve continuity of care and strengthen the resilience of health systems throughout sub‐Saharan Africa, future analyses should investigate how 2wT could be integrated with other comprehensive HIV prevention and digital health initiatives. To further support evidence‐based decision‐making, systematic reviews and meta‐analyses are recommended to quantify the effectiveness, cost‐effectiveness, and impact of 2wT interventions across diverse contexts.

## 6 Conclusion

The data from the included studies suggests that the 2wT intervention may support post‐operative VMMC care in sub‐Saharan African settings. Available findings suggest that 2wT is a potentially scalable approach for post‐VMMC follow‐up, as it reduces clinic visits while maintaining reported quality of care and helps in the identification of adverse events. The intervention was generally reported as user‐friendly, particularly in studies from South Africa and Zimbabwe, and may help reduce time and transport‐related burdens from clinic visits for patients. Overall, this scoping review highlights 2wT as a potentially useful mHealth approach for post‐operative care after VMMC, while underscoring the need for further research to assess its scalability and integration into broader health systems in sub‐Saharan Africa.

## Author Contributions

**Calsile Makhele:** conceptualization, methodology, writing – original draft, formal analysis, writing – review and editing, visualization. **Evans Duah:** visualization, supervision, conceptualization, methodology, writing – review and editing. **Smangaliso Morerwa:** methodology, data curation. **Kuhlula Maluleke:** conceptualization, methodology, formal analysis, validation, supervision, writing – review and editing.

## Funding

The authors have nothing to report.

## Ethics Statement

All authors have read and approved the final version of the manuscript. Calsile Makhele had full access to all the data in this study and takes complete responsibility for the integrity of the data and the accuracy of the data analysis.

## Conflicts of Interest

The authors declare no conflicts of interest.

## Transparency Statement

I Calsile Makhele, affirms that this manuscript is an honest, accurate, and transparent account of the study being reported; that no important aspects of the study have been omitted; and that any discrepancies from the study as planned have been explained.

## Supporting information

Supporting File 1

Supporting File 2

Supporting File 3

## Data Availability

The data that supports the findings of this study are available in the supporting material of this article. All data have been provided as supporting materials.
