# Supplementary material for: Reported Effectiveness of a Text‐Based Post‐Operative Care Intervention After Voluntary Medical Male Circumcision (VMMC) to Improve Quality of Care and Adverse Events Identification in Sub‐Saharan Africa: A Scoping Review
Source: Health Sci Rep. 2026 Jul 4;9(7):e72752. doi: 10.1002/hsr2.72752 (PMC13332858; doi:10.1002/hsr2.72752)
Supplement: Supplementary file 1 — Supporting File 1 [file HSR2-9-e72752-s001.docx]

**SCOPING REVIEW**

**Name of Principal Investigator:** Calsile Makhele

**Name of Supervisor:** Dr Kuhlula Maluleke

**Department:**

**Title of Review:** A Text-Based Post-Operative Intervention in Voluntary Medical Male Circumcision (VMMC) to Improve Quality of Care and Adverse Events Identification in Sub-Saharan Africa: A Scoping Review

**Research question**: What is the effectiveness of 2wT mHealth intervention in improving the quality of patient care and early identification of adverse events in VMMC post-operative care?

**PERIOD**: 2010 - 2025

| **CONCEPT 1** | **CONCEPT 2** | **CONCEPT 3** | **CONCEPT 4** | **CONCEPT 5** | **CONCEPT 6** |
| --- | --- | --- | --- | --- | --- |
| “Voluntary Medical Male Circumcision” | “2-way texting” | “Post-operative care” | “Quality of care” | “Feasibility” | “Sub-Saharan Africa” |
| “VMMC” | “2wT” | “Follow-up care” | “Healthcare quality” | “acceptability” | “SSA” |
| “Male circumcision” | “Interactive SMS’ | “Post-surgical care” | “Service delivery” | “usability” | “Low and middle-income countries” |
| “Medical circumcision” | “Mobile texting” | “Post-surgical education” | “Continuity of care” | “adoption” | “LMICs” |
| “Circumcised men” | “Text messaging” | “Surgical follow-up” | “Patient safety” | “implementation” | “Africa south of the Sahara” |
| “HIV prevention” | “mHealth” | “After care” | “Timely care” | “Barriers and facilitators” | “Resourced limited settings” |
|  | “Digital health” | “Post circumcision complications” |  | “scalability” |  |
|  | “SMS’ | “Patient safety” |  | “sustainability” |  |
|  | “Text-based follow-up” | AE management |  |  |  |
|  |  | Post-VMMC care |  |  |  |
|  |  | Adverse events |  |  |  |

**General strategy**:

("Voluntary Medical Male Circumcision" OR VMMC OR "male circumcision" OR "medical circumcision" OR "circumcised men" OR "HIV prevention" OR “Circumcised males”)

AND

("2-way texting" OR "two-way texting" OR 2wT OR "interactive SMS" OR "mobile texting" OR "text messaging" OR mHealth OR "mobile health" OR "SMS follow-up" OR "text-based follow-up" OR "digital health" OR “Interactive messaging” OR “Patient engagement” OR “Follow-up system” OR “Text-based telehealth”)

AND

(“quality of care” OR “healthcare quality” OR “service delivery” OR “continuity of care” OR “patient safety” OR “timely care” OR “Patient care” OR “Adverse events” OR “Post-operative care” OR “Post-VMMC care” OR “AE management”)

AND

(Intervention OR Initiative OR Program OR innovation )

AND

("low- and middle-income countries" OR LMIC* OR "developing countries" OR "resource-limited settings" OR "Sub-Saharan Africa" OR SSA OR Afghanistan OR Angola OR Algeria OR Bangladesh OR Benin OR Bhutan OR Bolivia OR "Burkina Faso" OR Burundi OR "Cabo Verde" OR "Cape Verde" OR Cambodia OR Cameroon OR Comoros OR "Republic of the Congo" OR "Democratic Republic of the Congo" OR "Côte d’Ivoire" OR "Côte d'Ivoire" OR "Ivory Coast" OR Djibouti OR Egypt OR "El Salvador" OR "Equatorial Guinea" OR Eritrea OR Eswatini OR Swaziland OR Ethiopia OR Gabon OR Gambia OR Ghana OR Guinea OR "Guinea-Bissau" OR Haiti OR Honduras OR India OR Indonesia OR Iran OR Kenya OR Kiribati OR "Kyrgyz Republic" OR "Lao PDR" OR Lesotho OR Liberia OR Madagascar OR Malawi OR Mali OR Mauritania OR Mauritius OR Micronesia OR Mongolia OR Morocco OR Mozambique OR Myanmar OR Namibia OR Nepal OR Nicaragua OR Niger OR Nigeria OR Pakistan OR "Papua New Guinea" OR Philippines OR "São Tomé and Príncipe" OR Senegal OR Seychelles OR "Sierra Leone" OR Somalia OR "South Africa" OR "South Sudan" OR Sudan OR "Syrian Arab Republic" OR Tajikistan OR Tanzania OR "Timor-Leste" OR Togo OR Tunisia OR Ukraine OR Uzbekistan OR Vanuatu OR Vietnam OR Zambia OR Zimbabwe OR "Sri Lanka")

| **DATABASE** | **STRATEGY** | **RESULTS** |
| --- | --- | --- |
| EBSCOhost: MEDLINE, CINAHL, Health Source: Nursing/Academic Edition, Health Source - Consumer Edition, Academic Complete, Africa-Wide Information  2025/08/14 | ("Voluntary Medical Male Circumcision" OR VMMC OR "male circumcision" OR "medical circumcision" OR "circumcised men" OR "HIV prevention") AND ("2-way texting" OR "two-way texting" OR 2wT OR "interactive SMS" OR "mobile texting" OR "text messaging" OR mHealth OR "mobile health" OR "SMS follow-up" OR "text-based follow-up" OR "digital health") AND ("adverse event identification" OR "AE detection" OR "complication monitoring" OR "surgical complications" OR "clinical alerts" OR "health event detection") AND ("quality of care" OR "healthcare quality" OR "service delivery" OR "continuity of care" OR "patient safety" OR "timely care") AND ("feasibility" OR "acceptability" OR "usability" OR "adoption" OR "implementation" OR "barriers and facilitators" OR "scalability") AND ("low- and middle-income countries" OR LMIC* OR "developing countries" OR "resource-limited settings" OR "Sub-Saharan Africa" OR SSA OR Afghanistan OR Angola OR Algeria OR Bangladesh OR Benin OR Bhutan OR Bolivia OR "Burkina Faso" OR Burundi OR "Cabo Verde" OR "Cape Verde" OR Cambodia OR Cameroon OR Comoros OR "Republic of the Congo" OR "Democratic Republic of the Congo" OR "Côte d’Ivoire" OR "Côte d'Ivoire" OR "Ivory Coast" OR Djibouti OR Egypt OR "El Salvador" OR "Equatorial Guinea" OR Eritrea OR Eswatini OR Swaziland OR Ethiopia OR Gabon OR Gambia OR Ghana OR Guinea OR "Guinea-Bissau" OR Haiti OR Honduras OR India OR Indonesia OR Iran OR Kenya OR Kiribati OR "Kyrgyz Republic" OR "Lao PDR" OR Lesotho OR Liberia OR Madagascar OR Malawi OR Mali OR Mauritania OR Mauritius OR Micronesia OR Mongolia OR Morocco OR Mozambique OR Myanmar OR Namibia OR Nepal OR Nicaragua OR Niger OR Nigeria OR Pakistan OR "Papua New Guinea" OR Philippines OR "São Tomé and Príncipe" OR Senegal OR Seychelles OR "Sierra Leone" OR Somalia OR "South Africa" OR "South Sudan" OR Sudan OR "Syrian Arab Republic" OR Tajikistan OR Tanzania OR "Timor-Leste" OR Togo OR Tunisia OR Ukraine OR Uzbekistan OR Vanuatu OR Vietnam OR Zambia OR Zimbabwe OR "Sri Lanka") | [Link](https://research-ebsco-com.uplib.idm.oclc.org/c/ei2kms/search/results?q=TX%20(%22Voluntary%20Medical%20Male%20Circumcision%22%20OR%20VMMC%20OR%20%22male%20circumcision%22%20OR%20%22medical%20circumcision%22%20OR%20%22circumcised%20men%22%20OR%20%22HIV%20prevention%22%20OR%20%E2%80%9CCircumcised%20males%E2%80%9D)%20AND%20TX%20(%222-way%20texting%22%20OR%20%22two-way%20texting%22%20OR%202wT%20OR%20%22interactive%20SMS%22%20OR%20%22mobile%20texting%22%20OR%20%22text%20messaging%22%20OR%20mHealth%20OR%20%22mobile%20health%22%20OR%20%22SMS%20follow-up%22%20OR%20%22text-based%20follow-up%22%20OR%20%22digital%20health%22%20OR%20%E2%80%9CInteractive%20messaging%E2%80%9D%20OR%20%E2%80%9CPatient%20engagement%E2%80%9D%20OR%20%E2%80%9CFollow-up%20system%E2%80%9D%20OR%20%E2%80%9CText-based%20telehealth%E2%80%9D)%20AND%20TX%20(Intervention%20OR%20Initiative%20OR%20Program%20OR%20innovation)%20AND%20TX%20(%E2%80%9Cquality%20of%20care%E2%80%9D%20OR%20%E2%80%9Chealthcare%20quality%E2%80%9D%20OR%20%E2%80%9Cservice%20delivery%E2%80%9D%20OR%20%E2%80%9Ccontinuity%20of%20care%E2%80%9D%20OR%20%E2%80%9Cpatient%20safety%E2%80%9D%20OR%20%E2%80%9Ctimely%20care%E2%80%9D%20OR%20%E2%80%9CPatient%20care%E2%80%9D%20OR%20%E2%80%9CAdverse%20events%E2%80%9D%20OR%20%E2%80%9CPost-operative%20care%E2%80%9D%20OR%20%E2%80%9CPost-VMMC%20care%E2%80%9D%20OR%20%E2%80%9CAE%20management%E2%80%9D)%20AND%20TX%20(%22low-%20and%20middle-income%20countries%22%20OR%20LMIC*%20OR%20%22developing%20countries%22%20OR%20%22resource-limited%20settings%22%20OR%20%22Sub-Saharan%20Africa%22%20OR%20SSA%20OR%20Afghanistan%20OR%20Angola%20OR%20Algeria%20OR%20Bangladesh%20OR%20Benin%20OR%20Bhutan%20OR%20Bolivia%20OR%20%22Burkina%20Faso%22%20OR%20Burundi%20OR%20%22Cabo%20Verde%22%20OR%20%22Cape%20Verde%22%20OR%20Cambodia%20OR%20Cameroon%20OR%20Comoros%20OR%20%22Republic%20of%20the%20Congo%22%20OR%20%22Democratic%20Republic%20of%20the%20Congo%22%20OR%20%22C%C3%B4te%20d%E2%80%99Ivoire%22%20OR%20%22C%C3%B4te%20d%27Ivoire%22%20OR%20%22Ivory%20Coast%22%20OR%20Djibouti%20OR%20Egypt%20OR%20%22El%20Salvador%22%20OR%20%22Equatorial%20Guinea%22%20OR%20Eritrea%20OR%20Eswatini%20OR%20Swaziland%20OR%20Ethiopia%20OR%20Gabon%20OR%20Gambia%20OR%20Ghana%20OR%20Guinea%20OR%20%22Guinea-Bissau%22%20OR%20Haiti%20OR%20Honduras%20OR%20India%20OR%20Indonesia%20OR%20Iran%20OR%20Kenya%20OR%20Kiribati%20OR%20%22Kyrgyz%20Republic%22%20OR%20%22Lao%20PDR%22%20OR%20Lesotho%20OR%20Liberia%20OR%20Madagascar%20OR%20Malawi%20OR%20Mali%20OR%20Mauritania%20OR%20Mauritius%20OR%20Micronesia%20OR%20Mongolia%20OR%20Morocco%20OR%20Mozambique%20OR%20Myanmar%20OR%20Namibia%20OR%20Nepal%20OR%20Nicaragua%20OR%20Niger%20OR%20Nigeria%20OR%20Pakistan%20OR%20%22Papua%20New%20Guinea%22%20OR%20Philippines%20OR%20%22S%C3%A3o%20Tom%C3%A9%20and%20Pr%C3%ADncipe%22%20OR%20Senegal%20OR%20Seychelles%20OR%20%22Sierra%20Leone%22%20OR%20Somalia%20OR%20%22South%20Africa%22%20OR%20%22South%20Sudan%22%20OR%20Sudan%20OR%20%22Syrian%20Arab%20Republic%22%20OR%20Tajikistan%20OR%20Tanzania%20OR%20%22Timor-Leste%22%20OR%20Togo%20OR%20Tunisia%20OR%20Ukraine%20OR%20Uzbekistan%20OR%20Vanuatu%20OR%20Vietnam%20OR%20Zambia%20OR%20Zimbabwe%20OR%20%22Sri%20Lanka%22)&autocorrect=y&db=a9h%2Cawn%2Ccin20%2Chxh%2Chch%2Ccmedm&expanders=fullText&isDashboardExpanded=true&limiters=RV%3AY%2CDT1%3A2015-08-22%2F2025-08-22&qm=W3sidHlwZSI6ImZpZWxkIiwidmFsdWUiOiJcIlZvbHVudGFyeSBNZWRpY2FsIE1hbGUgQ2lyY3VtY2lzaW9uXCIgT1IgVk1NQyBPUiBcIm1hbGUgY2lyY3VtY2lzaW9uXCIgT1IgXCJtZWRpY2FsIGNpcmN1bWNpc2lvblwiIE9SIFwiY2lyY3VtY2lzZWQgbWVuXCIgT1IgXCJISVYgcHJldmVudGlvblwiIE9SIOKAnENpcmN1bWNpc2VkIG1hbGVz4oCdIiwiY29kZSI6IlRYIn0seyJ0eXBlIjoibG9naWMiLCJ2YWx1ZSI6IkFORCJ9LHsidHlwZSI6ImZpZWxkIiwidmFsdWUiOiJcIjItd2F5IHRleHRpbmdcIiBPUiBcInR3by13YXkgdGV4dGluZ1wiIE9SIDJ3VCBPUiBcImludGVyYWN0aXZlIFNNU1wiIE9SIFwibW9iaWxlIHRleHRpbmdcIiBPUiBcInRleHQgbWVzc2FnaW5nXCIgT1IgbUhlYWx0aCBPUiBcIm1vYmlsZSBoZWFsdGhcIiBPUiBcIlNNUyBmb2xsb3ctdXBcIiBPUiBcInRleHQtYmFzZWQgZm9sbG93LXVwXCIgT1IgXCJkaWdpdGFsIGhlYWx0aFwiIE9SIOKAnEludGVyYWN0aXZlIG1lc3NhZ2luZ%2BKAnSBPUiDigJxQYXRpZW50IGVuZ2FnZW1lbnTigJ0gT1Ig4oCcRm9sbG93LXVwIHN5c3RlbeKAnSBPUiDigJxUZXh0LWJhc2VkIHRlbGVoZWFsdGjigJ0iLCJjb2RlIjoiVFgifSx7InR5cGUiOiJsb2dpYyIsInZhbHVlIjoiQU5EIn0seyJ0eXBlIjoiZmllbGQiLCJ2YWx1ZSI6IkludGVydmVudGlvbiBPUiBJbml0aWF0aXZlIE9SIFByb2dyYW0gT1IgaW5ub3ZhdGlvbiIsImNvZGUiOiJUWCJ9LHsidHlwZSI6ImxvZ2ljIiwidmFsdWUiOiJBTkQifSx7InR5cGUiOiJmaWVsZCIsInZhbHVlIjoi4oCccXVhbGl0eSBvZiBjYXJl4oCdIE9SIOKAnGhlYWx0aGNhcmUgcXVhbGl0eeKAnSBPUiDigJxzZXJ2aWNlIGRlbGl2ZXJ54oCdIE9SIOKAnGNvbnRpbnVpdHkgb2YgY2FyZeKAnSBPUiDigJxwYXRpZW50IHNhZmV0eeKAnSBPUiDigJx0aW1lbHkgY2FyZeKAnSBPUiDigJxQYXRpZW50IGNhcmXigJ0gT1Ig4oCcQWR2ZXJzZSBldmVudHPigJ0gT1Ig4oCcUG9zdC1vcGVyYXRpdmUgY2FyZeKAnSBPUiDigJxQb3N0LVZNTUMgY2FyZeKAnSBPUiDigJxBRSBtYW5hZ2VtZW504oCdIiwiY29kZSI6IlRYIn0seyJ0eXBlIjoibG9naWMiLCJ2YWx1ZSI6IkFORCJ9LHsidHlwZSI6ImZpZWxkIiwidmFsdWUiOiJcImxvdy0gYW5kIG1pZGRsZS1pbmNvbWUgY291bnRyaWVzXCIgT1IgTE1JQyogT1IgXCJkZXZlbG9waW5nIGNvdW50cmllc1wiIE9SIFwicmVzb3VyY2UtbGltaXRlZCBzZXR0aW5nc1wiIE9SIFwiU3ViLVNhaGFyYW4gQWZyaWNhXCIgT1IgU1NBIE9SIEFmZ2hhbmlzdGFuIE9SIEFuZ29sYSBPUiBBbGdlcmlhIE9SIEJhbmdsYWRlc2ggT1IgQmVuaW4gT1IgQmh1dGFuIE9SIEJvbGl2aWEgT1IgXCJCdXJraW5hIEZhc29cIiBPUiBCdXJ1bmRpIE9SIFwiQ2FibyBWZXJkZVwiIE9SIFwiQ2FwZSBWZXJkZVwiIE9SIENhbWJvZGlhIE9SIENhbWVyb29uIE9SIENvbW9yb3MgT1IgXCJSZXB1YmxpYyBvZiB0aGUgQ29uZ29cIiBPUiBcIkRlbW9jcmF0aWMgUmVwdWJsaWMgb2YgdGhlIENvbmdvXCIgT1IgXCJDw7R0ZSBk4oCZSXZvaXJlXCIgT1IgXCJDw7R0ZSBkJ0l2b2lyZVwiIE9SIFwiSXZvcnkgQ29hc3RcIiBPUiBEamlib3V0aSBPUiBFZ3lwdCBPUiBcIkVsIFNhbHZhZG9yXCIgT1IgXCJFcXVhdG9yaWFsIEd1aW5lYVwiIE9SIEVyaXRyZWEgT1IgRXN3YXRpbmkgT1IgU3dhemlsYW5kIE9SIEV0aGlvcGlhIE9SIEdhYm9uIE9SIEdhbWJpYSBPUiBHaGFuYSBPUiBHdWluZWEgT1IgXCJHdWluZWEtQmlzc2F1XCIgT1IgSGFpdGkgT1IgSG9uZHVyYXMgT1IgSW5kaWEgT1IgSW5kb25lc2lhIE9SIElyYW4gT1IgS2VueWEgT1IgS2lyaWJhdGkgT1IgXCJLeXJneXogUmVwdWJsaWNcIiBPUiBcIkxhbyBQRFJcIiBPUiBMZXNvdGhvIE9SIExpYmVyaWEgT1IgTWFkYWdhc2NhciBPUiBNYWxhd2kgT1IgTWFsaSBPUiBNYXVyaXRhbmlhIE9SIE1hdXJpdGl1cyBPUiBNaWNyb25lc2lhIE9SIE1vbmdvbGlhIE9SIE1vcm9jY28gT1IgTW96YW1iaXF1ZSBPUiBNeWFubWFyIE9SIE5hbWliaWEgT1IgTmVwYWwgT1IgTmljYXJhZ3VhIE9SIE5pZ2VyIE9SIE5pZ2VyaWEgT1IgUGFraXN0YW4gT1IgXCJQYXB1YSBOZXcgR3VpbmVhXCIgT1IgUGhpbGlwcGluZXMgT1IgXCJTw6NvIFRvbcOpIGFuZCBQcsOtbmNpcGVcIiBPUiBTZW5lZ2FsIE9SIFNleWNoZWxsZXMgT1IgXCJTaWVycmEgTGVvbmVcIiBPUiBTb21hbGlhIE9SIFwiU291dGggQWZyaWNhXCIgT1IgXCJTb3V0aCBTdWRhblwiIE9SIFN1ZGFuIE9SIFwiU3lyaWFuIEFyYWIgUmVwdWJsaWNcIiBPUiBUYWppa2lzdGFuIE9SIFRhbnphbmlhIE9SIFwiVGltb3ItTGVzdGVcIiBPUiBUb2dvIE9SIFR1bmlzaWEgT1IgVWtyYWluZSBPUiBVemJla2lzdGFuIE9SIFZhbnVhdHUgT1IgVmlldG5hbSBPUiBaYW1iaWEgT1IgWmltYmFid2UgT1IgXCJTcmkgTGFua2FcIiIsImNvZGUiOiJUWCJ9XQ%3D%3D&searchMode=all&searchSegment=all-results)  968 |
| Scopus  2025/07/18 | ( ALL ( "Voluntary Medical Male Circumcision" OR vmmc OR "male circumcision" OR "medical circumcision" OR "circumcised men" OR "HIV prevention" OR "Circumcised males" ) AND ALL ( "2-way texting" OR "two-way texting" OR 2wt OR "interactive SMS" OR "mobile texting" OR "text messaging" OR mhealth OR "mobile health" OR "SMS follow-up" OR "text-based follow-up" OR "digital health" OR "Interactive messaging" OR "Patient engagement" OR "Follow-up system" OR "Text-based telehealth" ) AND ALL ( "quality of care" OR "healthcare quality" OR "service delivery" OR "continuity of care" OR "patient safety" OR "timely care" OR "Patient care" OR "Adverse events" OR "Post-operative care" OR "Post-VMMC care" OR "AE management" ) AND ALL ( intervention OR initiative OR program OR innovation ) AND ALL ( "low- and middle-income countries" OR lmic* OR "developing countries" OR "resource-limited settings" OR "Sub-Saharan Africa" OR ssa OR afghanistan OR angola OR algeria OR bangladesh OR benin OR bhutan OR bolivia OR "Burkina Faso" OR burundi OR "Cabo Verde" OR "Cape Verde" OR cambodia OR cameroon OR comoros OR "Republic of the Congo" OR "Democratic Republic of the Congo" OR "Côte d’Ivoire" OR "Côte d'Ivoire" OR "Ivory Coast" OR djibouti OR egypt OR "El Salvador" OR "Equatorial Guinea" OR eritrea OR eswatini OR swaziland OR ethiopia OR gabon OR gambia OR ghana OR guinea OR "Guinea-Bissau" OR haiti OR honduras OR india OR indonesia OR iran OR kenya OR kiribati OR "Kyrgyz Republic" OR "Lao PDR" OR lesotho OR liberia OR madagascar OR malawi OR mali OR mauritania OR mauritius OR micronesia OR mongolia OR morocco OR mozambique OR myanmar OR namibia OR nepal OR nicaragua OR niger OR nigeria OR pakistan OR "Papua New Guinea" OR philippines OR "São Tomé and Príncipe" OR senegal OR seychelles OR "Sierra Leone" OR somalia OR "South Africa" OR "South Sudan" OR sudan OR "Syrian Arab Republic" OR tajikistan OR tanzania OR "Timor-Leste" OR togo OR tunisia OR ukraine OR uzbekistan OR vanuatu OR vietnam OR zambia OR zimbabwe OR "Sri Lanka" ) ) AND PUBYEAR > 2014 AND PUBYEAR < 2026 | [Link](https://www-scopus-com.uplib.idm.oclc.org/results/results.uri?st1=%22Voluntary+Medical+Male+Circumcision%22+OR+VMMC+OR+%22male+circumcision%22+OR+%22medical+circumcision%22+OR+%22circumcised+men%22+OR+%22HIV+prevention%22+OR+%22Circumcised+males%22&st2=%222-way+texting%22+OR+%22two-way+texting%22+OR+2wT+OR+%22interactive+SMS%22+OR+%22mobile+texting%22+OR+%22text+messaging%22+OR+mHealth+OR+%22mobile+health%22+OR+%22SMS+follow-up%22+OR+%22text-based+follow-up%22+OR+%22digital+health%22+OR+%22Interactive+messaging%22+OR+%22Patient+engagement%22+OR+%22Follow-up+system%22+OR+%22Text-based+telehealth%22&limit=10&origin=resultslist&sort=plf-f&src=s&sot=b&sdt=cl&sessionSearchId=c0a598de946566e9e86b8dff4a4900ae&yearFrom=2015&yearTo=2025)  1678 |
| PubMed  2025/07/18 | (((("Voluntary Medical Male Circumcision" OR VMMC OR "male circumcision" OR "medical circumcision" OR "circumcised men" OR "HIV prevention" OR "Circumcised males"[MeSH Terms]) AND ("2-way texting" OR "two-way texting" OR 2wT OR "interactive SMS" OR "mobile texting" OR "text messaging" OR mHealth OR "mobile health" OR "SMS follow-up" OR "text-based follow-up" OR "digital health" OR "Interactive messaging" OR "Patient engagement" OR "Follow-up system" OR "Text-based telehealth")) AND ("quality of care" OR "healthcare quality" OR "service delivery" OR "continuity of care" OR "patient safety" OR "timely care" OR "Patient care" OR "Adverse events" OR "Post-operative care" OR "Post-VMMC care" OR "AE management")) AND (Intervention OR Initiative OR Program OR innovation)) AND ("low- and middle-income countries" OR LMIC* OR "developing countries" OR "resource-limited settings" OR "Sub-Saharan Africa" OR SSA OR Afghanistan OR Angola OR Algeria OR Bangladesh OR Benin OR Bhutan OR Bolivia OR "Burkina Faso" OR Burundi OR "Cabo Verde" OR "Cape Verde" OR Cambodia OR Cameroon OR Comoros OR "Republic of the Congo" OR "Democratic Republic of the Congo" OR "Côte d’Ivoire" OR "Côte d'Ivoire" OR "Ivory Coast" OR Djibouti OR Egypt OR "El Salvador" OR "Equatorial Guinea" OR Eritrea OR Eswatini OR Swaziland OR Ethiopia OR Gabon OR Gambia OR Ghana OR Guinea OR "Guinea-Bissau" OR Haiti OR Honduras OR India OR Indonesia OR Iran OR Kenya OR Kiribati OR "Kyrgyz Republic" OR "Lao PDR" OR Lesotho OR Liberia OR Madagascar OR Malawi OR Mali OR Mauritania OR Mauritius OR Micronesia OR Mongolia OR Morocco OR Mozambique OR Myanmar OR Namibia OR Nepal OR Nicaragua OR Niger OR Nigeria OR Pakistan OR "Papua New Guinea" OR Philippines OR "São Tomé and Príncipe" OR Senegal OR Seychelles OR "Sierra Leone" OR Somalia OR "South Africa" OR "South Sudan" OR Sudan OR "Syrian Arab Republic" OR Tajikistan OR Tanzania OR "Timor-Leste" OR Togo OR Tunisia OR Ukraine OR Uzbekistan OR Vanuatu OR Vietnam OR Zambia OR Zimbabwe OR "Sri Lanka") Filters: in the last 10 years | [Link](https://pubmed-ncbi-nlm-nih-gov.uplib.idm.oclc.org/?term=longquery712a076da7ae1708fbd0&filter=datesearch.y_10)  33 |
| Web of Science  (2025/07/18) | "Voluntary Medical Male Circumcision" OR VMMC OR "male circumcision" OR "medical circumcision" OR "circumcised men" OR "HIV prevention" (Topic) and "2-way texting" OR "two-way texting" OR 2wT OR "interactive SMS" OR "mobile texting" OR "text messaging" OR mHealth OR "mobile health" OR "SMS follow-up" OR "text-based follow-up" OR "digital health" (Topic) and "adverse event identification" OR "AE detection" OR "complication monitoring" OR "surgical complications" OR "clinical alerts" OR "health event detection" (Topic) and “quality of care” OR “healthcare quality” OR “service delivery” OR “continuity of care” OR “patient safety” OR “timely care” (Topic) and "feasibility" OR "acceptability" OR "usability" OR "adoption" OR "implementation" OR "barriers and facilitators" OR "scalability" (Topic) and "low- and middle-income countries" OR LMIC* OR "developing countries" OR "resource-limited settings" OR "Sub-Saharan Africa" OR SSA OR Afghanistan OR Angola OR Algeria OR Bangladesh OR Benin OR Bhutan OR Bolivia OR "Burkina Faso" OR Burundi OR "Cabo Verde" OR "Cape Verde" OR Cambodia OR Cameroon OR Comoros OR "Republic of the Congo" OR "Democratic Republic of the Congo" OR "Côte d’Ivoire" OR "Côte d'Ivoire" OR "Ivory Coast" OR Djibouti OR Egypt OR "El Salvador" OR "Equatorial Guinea" OR Eritrea OR Eswatini OR Swaziland OR Ethiopia OR Gabon OR Gambia OR Ghana OR Guinea OR "Guinea-Bissau" OR Haiti OR Honduras OR India OR Indonesia OR Iran OR Kenya OR Kiribati OR "Kyrgyz Republic" OR "Lao PDR" OR Lesotho OR Liberia OR Madagascar OR Malawi OR Mali OR Mauritania OR Mauritius OR Micronesia OR Mongolia OR Morocco OR Mozambique OR Myanmar OR Namibia OR Nepal OR Nicaragua OR Niger OR Nigeria OR Pakistan OR "Papua New Guinea" OR Philippines OR "São Tomé and Príncipe" OR Senegal OR Seychelles OR "Sierra Leone" OR Somalia OR "South Africa" OR "South Sudan" OR Sudan OR "Syrian Arab Republic" OR Tajikistan OR Tanzania OR "Timor-Leste" OR Togo OR Tunisia OR Ukraine OR Uzbekistan OR Vanuatu OR Vietnam OR Zambia OR Zimbabwe OR "Sri Lanka" (Topic) and 2010-2025 (Year Published) and Preprint Citation Index (Exclude – Database) | [Link](https://www-webofscience-com.uplib.idm.oclc.org/wos/alldb/summary/e1776d07-1aed-46f0-8ed0-e2b36f76c7d3-01754e6df2/relevance/1)  188 |

| Citation manual searching | 2 |
| --- | --- |
