# Supplementary material for: Reported Effectiveness of a Text‐Based Post‐Operative Care Intervention After Voluntary Medical Male Circumcision (VMMC) to Improve Quality of Care and Adverse Events Identification in Sub‐Saharan Africa: A Scoping Review
Source: Health Sci Rep. 2026 Jul 4;9(7):e72752. doi: 10.1002/hsr2.72752 (PMC13332858; doi:10.1002/hsr2.72752)
Supplement: Supplementary file 2 — Supporting File 2 [file HSR2-9-e72752-s003.docx]

| Author & Year of publication | Title | Aim of the study | Study population | Study location (country) | Study design | Type of text-based intervention | Effectiveness/Impact of the text-based intervention | Usability of the intervention | How intervention improves quality of care | How the intervention identifies adverse events | Cost-effectiveness of the intervention | Other significant findings |
| --- | --- | --- | --- | --- | --- | --- | --- | --- | --- | --- | --- | --- |
| Fabens et al. 2024 | WhatsApp Versus SMS for 2-Way, Text-Based Follow-Up After Voluntary Medical Male Circumcision in South Africa: Exploration of Messaging Platform Choice | 1. To explore 2wT client preferences between WhatsApp or SMS including client satisfaction  2. To examine response rates (participation) by SMS and WhatsApp; 3. to gather feedback from the 2wT implementation team on the WhatsApp approach for 2wT | VMMC males who opted into the 2wT-based follow-up approach | South Africa (Urban and rural) | Quasi-experimental, 4-step, stepped wedge study: comparison of WhatsApp to SMS | Two-way-texting | For effectiveness, males responded similarly via WhatsApp and SMS with 70% of participants responding within 3 days after VMMC and 79% responding within 14 days post VMMC. | 53% of participants chose WhatsApp as their platform (P=.38) | 98% were happy with the way they were followed up: Clients using both platforms responded to 2wT interactive prompts, demonstrating similar effectiveness in engaging clients in follow-up |  |  | Challenge: 2wT team members believed WhatsApp limited the automated message content, language choices, and inclusivity as compared with the SMS-based 2wT approach Clients appear evenly split between SMS and WhatsApp. |
| Unsworth et al.2025 | Expanding two-way texting for post-operative follow-up: A cost analysis of the implementation and scale-up in routine voluntary medical male circumcision settings in South Africa | To assess the feasibility of 2wT expansion in routine practice and provide updated estimates on the costs associated with the use of 2wT in routine practice and during VMMC campaigns | Males aged 15 years or older who underwent VMMC | South Africa (Rural and urban) | Activity-based cost analysis | Two-way texting (2wT) | 2wT increase cost savings while dramatically reducing the burden of in-person visits on patients and clinics |  | 2wT quality care improved | AE ascertainment increased while loss to follow-up decreased. | 2wT saves $0.29/client with 60% 2wT enrolment and saves $0.46/client with 80% 2wT enrolment when scaled. | Routine clients had a higher rate of loss to follow-up (LTFU) at 8% compared to 2wT participants at 4% |
| Feldacker et al. 2023 | Expanding the Evidence on the Safety and Efficiency of 2-Way Text Messaging Telehealth for Voluntary Medical Male Circumcision Follow-up Compared with In-Person Reviews: Randomized Controlled Trial in Rural and Urban South Africa. | To determine whether 2wT improves adverse event (AE) ascertainment, the quality of follow-up after VMMC while reducing health care workers€™ workload. | Adult men who underwent VMMC with cell phones | South Africa (urban and rural) | Randomized Controlled Trial (RCT) | 2-way texting (2wT) | 2wT approach reduced unnecessary postoperative visits by 84.8% |  | 2wT provides quality VMMC follow-up | Cumulative AEs were identified in 2.3% (95% CI 1.3-4.1) of 2wT participants and 1.0% (95% CI 0.4-2.3) of control participants with a difference of (P=.13) AE rates |  | 2wT approach also significantly reduced the follow-up visit workload, improving efficiency |
| Fabens et al.2025 | When evidence is not enough: A qualitative exploration of healthcare workers' perspectives on expansion of two-way texting (2wT) for post- circumcision follow-up in South Africa. | To explore provider perspectives on 2wT :  1) implementation (RE-AIM) at the clinician and organizational levels to identify features for improved client, provider, and organizational experience  2) internal context (PRISM) to understand how to effectively work within the routine context of staffing structures and client preferences  3) external context (PRISM) that may influence 2wT maintenance such as guidelines and targets | Staff members involved in 2wT implementation: management, clinicians, data officials (M&E team) and support staff involved in 2wT scale-up | South Africa (urban and rural) | Qualitative study | Two-way-texting (2wT) | 2wT increases timely communication between clients and providers; fills potential gaps in client education; improves follow-up verification; and strengthens reporting. | A 10-year-old would be able to use it [2wT], because it asks you a question you either answer with zero or you answer with a one. 2wT dashboards has complete client data which further builds support for 2wTâ€™s effect on data quality | 2wT augments, not replaces, in-person visits: If a client is alone, they are more comfortable when they€™re in their own space so they can actually text a client would rather be there and be ill [rather than] coming in | Although the efficiency and safety benefits of 2wT were noted most participants (3 clinicians, 4 M&E staff and 1 manager) who felt 2wT increased their ability to identify adverse events |  | 2wT obstacles appear more influential than  perceived benefits, reducing buy-in and momentum to scale: perceptions of increased  workload, lack of site champions and lack of an enabling policy environment(providers lacked confidence that 2wT could ensure quality care with referral and tracing safeguards and frequent staff turnover). participants noted confusion caused by inconsistencies between external policies and 2wT as well as follow-up requirements which creates a potential false workload: I know the Department of Health does not like 2wT |
| Marongwe et al. 2022 | Transitioning a digital health innovation from research to routine practice: Two-way texting for male circumcision follow-up in Zimbabwe | To compare between 2wT from RCT and routine scale-up MC practices (safety and efficiency outcomes) | Adult males over age 18 that received an MC in the ZAZIC routine VMMC program | Zimbabwe (urban and rural) | A 3-tier system was developed to take 2wT from RCT to scale | Two-way texting | HCW workload was dramatically lower with 2wT: only 1.1% of 2wT versus 67.0% of routine follow-up men had in person |  | 2wT includes a daily care prompt to encourage men to observe their healing and engage directly with an MC nurse to ask questions or request a call back. | Prevention of LTFU improved during 2wT at scale: only 0.8% of 2wT men had no contact on upscale compared to RCT at 6.9% | 2wT ascertained fewer AEs, with AEs rates decreasing from 1.9% (95% CI: 0.7, 3.6) to 0.08% (95% CI: 0.03, 2.0), respectively (p<0.001) | Despite the challenges of MC service delivery during the COVID-19 pandemic, 2wT scaled successfully from the RCT to routine settings. |
| Feldacker et al. 2020 | Usability and acceptability of a two-way texting intervention for post-operative follow-up for voluntary medical male circumcision in Zimbabwe | To explore 2wT usability and acceptability among 2wT patients and healthcare providers focusing on understanding how providers and patients interacted with the 2wT system and obtaining insights into the obstacles and facilitators of system use | Men undergone circumcision | Zimbabwe (peri-urban and rural) | Mixed-methods study | Two-way texting | 2wT reduced their overall workload: reducing burnout on health care workers | Survey responses suggest that most clients felt confident, comfortable, satisfied, and safe with SMS follow-up | 2wT empowered men to engage in their own healing, assuring them that their clients were capable of identifying potential problems and seeking care when they wished rather than on a mandated schedule |  | 2wT would save men money by reducing transports costs, because texting only takes 5 cents compared to transport fees. | Messages reduce time and if there is a problem you are given time to communicate. It's just an efficient way of communicating |
| Feldacker et al. 2020 | Reducing Provider Workload While Preserving Patient Safety: A Randomized Control Trial Using 2-Way Texting for Postoperative Follow-up in Zimbabwe's Voluntary Medical Male Circumcision Program | 1. To determine if 2-way texting (2wT) can safely reduce MC follow-up visits. 2. To estimate the cost savings associated with 2wT over routine MC follow-up. 3. To assess the acceptability and feasibility of 2wT for further scale-up. | Men aged 18 years or older and underwent surgical Male circumcision | Zimbabwe (Rural and urban) | A Randomized Control Trial | 2-way texting (2wT) intervention | There is an 85% reduction in the workload over routinely scheduled visits | Response rates were high: 285 (78.7%) responded at least once before day 2; 326 (90.0%) responded at least once by day 7; and 334 men (92.5%) responded at least once over 13 days | For 48% of PAE texts, the nurse triaged the patient via text and did not refer for in-person review. For those referred for review, 9% did not return nor subsequently respond without concerns | An increase in reported AEs within the intervention arm of 1.04%: The increased ascertainment of AEs may also be the result of enhanced postoperative counselling and daily follow-up, critical components of the 2wT intervention |  | 2wT should be considered safe: 2wT appears safe for patients. 2wT ascertained and reported more AEs than routine care, suggesting that this follow-up method approximates active surveillance, improving the quality of patient care. |
| Day et al. 2023 | Centering Frontline Health Care Workers in Digital Innovation Design to Inform the Optimization of an App for Improved Male Circumcision Follow-up in South Africa: Qualitative Usability Study | To explore the usability, acceptability, and strengths of and suggestions for 2wT with HCWs involved in the RCT planning, implementation, and evaluation | Health care workers (HCWs) involved in 2wT implementation | South Africa (both urban and rural) | Qualitative study: using key informant interviews (KIIs) | Two-way-texting (2wT) | 2wT approach was reported to be beneficial not only to clients but also to HCWs by reducing their workload associated with daily client reviews | HCWs reported that the 2wT system is simple to use for HCWs | The 2wT system also offered benefits to client care in terms of infection prevention during the COVID-19 pandemic because patients did not have to come in and stand in the long queues |  | Time and cost savings was reported by the HCWs as a primary benefit for 2wT clients | Men also responded via 2wT over weekends, after hours, and on public holidays, creating work for the clinician outside routine clinic hours |
| Piennar et al. 2023 | 'I understood the texting process well'. Participant perspectives on usability and acceptability of SMS-based telehealth follow-up after voluntary medical male circumcision in South Africa | To evaluate 2wT usability and acceptability among 2wT arm participants to determine levels of MC client participation, perspective, and usefulness among these clients to inform 2wT expansion | Men who used 2wT to interact with an MC provider via SMS | South Africa (Both Urban and rural) | Quantitative study | Two-way-texting (2wT) intervention | most clients understood the 2wT process (63%), felt comfortable (98%), felt safe (92%) and would recommend this approach to their friends (87% | Men responded consistently to their daily texts with daily response rates ranging from 74% to 85% | 2wT provided remote clinical oversight for wound monitoring, offering a safe alternative: Men are typically reluctant to access healthcare in clinical settings, partially due to work or high mobility, resulting in late treatment seeking | The 2wT intervention enabled men to monitor and self-manage their healing process, with encouragement to seek early care for Potential-AEs (PAEs) | Majority of clients (60%) indicated that 2wT saved them time and money from attending in-person clinic visits | High response rates (88%) to daily messages indicated acceptability |
| Elkins et al. 2024 | "Endless opportunities": A qualitative exploration of facilitators and barriers to scale-up of two-way texting follow-up after voluntary medical male circumcision in Zimbabwe | To identify 2wT program strengths, challenges, and suggestions for 2wT scale up as part of routine ZAZIC VMMC services from the perspective of 2wT stakeholders | 2wT program partners: nurses, monitoring & evaluation teams, and technology partners | Zimbabwe, Kenya, Ghana, Uganda, and Nigeria (Mix of urban, peri-urban and rural) | Exploratory qualitative study | Two-way, text-based (2wT) intervention | 2wT improves follow-up data quality since the system both stores interactive messages and alerts nurses for timely documentation of referrals to care outcomes.  The Ministry really appreciated this two-way texting intervention and supported the scale up | The 2wT app is easy to use, making it attractive for healthcare worker: the simplicity of the app for HCW and client users was attributed to the 2wT co-design approach | 2wT improves quality of client follow-up care |  | 2wT program contributed to resource savings, both financial and human. | 2wT program was well received by clients, even though lack of available HCWs, technology challenges, client communication costs, and complexities in the transition to MoHCC remained a challenge |
| Babigumira et al. 2020 | Cost-effectiveness analysis of two-way texting for post-operative follow-up in Zimbabwe's voluntary medical male circumcision program | To assess the cost-effectiveness of 2wT in Zimbabwe VMMC program | Adult men over 18 years who underwent VMMC by dorsal slit | Zimbabwe (A mixture of rural and urban VMMC clinics operated by ZAZIC) | Randomized-controlled trial | Two-way texting (2wT) | 2wT was more effective than routine by reducing clinic visits, outreach visits and onset adverse events by increasing its management. | 2wT dominated standard of care in incremental analysis. |  | The 2wT intervention also increased AE ascertainment (as compared to the expected AE yield of 2%) by 50% (92% AE yield in 2wT compared to 42% AE yield in SoC). | The net impact was that 2wT reduced the mean costs of VMMC follow-up by $2.10. |  |
| Su et al. 2023 | Cost savings in male circumcision post-operative care using two-way text-based follow-up in rural and urban South Africa. | To compare 2wT and routine post-VMMC care costs in rural and urban South African settings | VMMC client using the 2wT | South Africa (Both rural and urban) | Activity-based costing (ABC) approach | two-way (2wT) follow-up | 2wT improves the quality of post-VMMC follow-up care at lower overall cost by providing an SMS-based mHealth option for clients with access to cell phones, encouraging visits when needed instead of compulsory visits on day 2 and/or day 7. |  |  | Daily SMS communication improved early detection of AEs and subsequent swift referral of those in need for in-person clinical visits. This triaging process led to identification of AEs earlier with less severity, likely averting costs of more severe AE | 2wT saved $3.56 per client as compared to routine care | Savings using the 2wT approach were higher in rural as compared to urban areas  “An important finding as the majority of VMMC program implementation occurs in rural areas. Quality assurance likely also benefits from 2wT improvements in verification of, and supervision for, quality post-operative care |
| Setswe et.al  2025 | Finding the balance between rigour and relevance: implementing adaptations to the implementation of a pragmatic randomised controlled trial of a two-way texting intervention for voluntary medical male circumcision in South Africa | To document adaptations that were made to the implementation of the two- way texting (2wT) randomised controlled trial (RCT) for voluntary medical male circumcision (VMMC) in South Africa and to provide a nuanced discussion on the differences between adaptations and fidelity in this context. | VMMC Men | South Africa (Rural and Urban Rural | Qualitative using- Framework for Reporting Adaptations and Modifications in Evidence- based Implementation Strategies (FRAME- IS) | Two-way-texting | reduced clinicians’ workload while increasing the intervention’s reach |  | team adapted the enrolment process to include collecting additional contact details to assist with tracing |  |  | fidelity and adaptation may not be opposing concepts; instead, there should be an exploration of how to achieve a balance between intervention fidelity and adaptation within interventions |
| Feldacker et.al  2025 | Strengthening evidence for text-based telehealth in post-operative care: A pragmatic study of the reach and effectiveness of two-way, text-based follow-up after voluntary medical male circumcision in South Africa | to determine if gains in adverse event (AE) identification and reduced follow-up visits could be maintained when 2wT was implemented in routine VMMC settings. | VMMC Men | South Africa (Rural and Urban Rural | pragmatic, stepped wedge design (SWD) study | (80%) responded via 2wT over 14 days, demonstrating engagement in post-operative care | 2wT follow-up approach ascertained nearly four times as many AEs as the routine, |  | reduced in-person visits dramatically, encouraging only those few with a desire or need to return for in-person reviews. | the AE ascertainment rate was higher among 2wT (0.60%) than SoC (0.13%) clients (p = 0.0018), demonstrating safety gains. |  |  |
